# Engineering *Paracoccus denitrificans* PD1222 for *Fusarium solani* cutinase-mediated biodegradation of poly(butylene adipate-co-terephthalate)

**DOI:** 10.1128/aem.00358-26

**Published:** 2026-06-18

**Authors:** Diego Martín-González, Carlos de la Fuente Tagarro, Raúl Muñoz, Sergio Bordel, Fernando Santos-Beneit

**Affiliations:** 1 Institute of Sustainable Processes, Valladolid, Spain; 2 Department of Chemical Engineering and Environmental Technology, School of Industrial Engineering, University of Valladolid 16782, Valladolid, Spain; 3 Department of Microbiology and Genetics, University of Salamanca 16779, Salamanca, Spain; University of Nebraska-Lincoln, Lincoln, Nebraska, USA

**Keywords:** *Paracoccus denitrificans *PD1222, poly(butylene adipate-co-terephthalate), depolymerization, *Fusarium solani*, cutinase, transformation, terephthalic acid, poly(3-hydroxybutyrate)

## Abstract

Poly(butylene adipate-co-terephthalate) (PBAT) is an aromatic–aliphatic copolyester widely used in packaging and consumer products. Its aromatic rings confer high resistance to hydrolysis, limiting biological degradation. To enhance PBAT biodegradation, we engineered *Paracoccus denitrificans* PD1222, a metabolically versatile and genetically tractable bacterium that can accumulate poly(3-hydroxybutyrate) (PHB). A novel plasmid (pV1) was constructed to express the broad-specificity cutinase FsCut under the constitutive *P*tuf promoter and fused to the PorG signal peptide for extracellular secretion. Using an optimized transformation protocol, we stably transformed *P. denitrificans* PD1222 with pV1, enabling secretion of active FsCut and efficient PBAT hydrolysis. In degradation assays, the engineered strain exhibited significantly higher depolymerization rates than the strain carrying the pV0 vector, used as a control. Furthermore, the FsCut-expressing strain accumulated 1.16-fold more PHB than the pV0 strain and exhibited degradation rates of PBAT 2.27-fold higher in enriched medium and 1.9-fold higher in defined mineral medium. These findings demonstrate that targeted expression of a secreted cutinase substantially improves PBAT degradation by *P. denitrificans*, supporting its potential as a microbial platform for plastic bioremediation.

**IMPORTANCE:**

The widespread use of poly(butylene adipate-co-terephthalate) (PBAT) is limited by its low hydrolytic degradation rate, resulting from its aromatic structure, which makes it highly resistant to biological degradation. Developing strategies to accelerate PBAT depolymerization is critical for advancing sustainable plastic waste management. In this study, a novel plasmid, pV1, was successfully constructed to enable the heterologous expression and extracellular secretion of the broad-specificity cutinase FsCut in *Paracoccus denitrificans* PD1222. The engineered strain effectively hydrolyzes PBAT, demonstrating the plasmid’s capability for correct synthesis and export of functional protein. Furthermore, the implementation of a newly developed bacterial transformation protocol in *P. denitrificans* represents a significant methodological advancement, reducing the time and complexity required compared with traditional conjugation approaches. Together, these findings highlight a dual achievement: improving the enzymatic degradation of a polymer with a low hydrolytic degradation rate and establishing an efficient genetic engineering strategy in a metabolically versatile bacterium, providing a promising platform for future bioremediation and biopolymer valorization studies.

## INTRODUCTION

**Fig 1:**
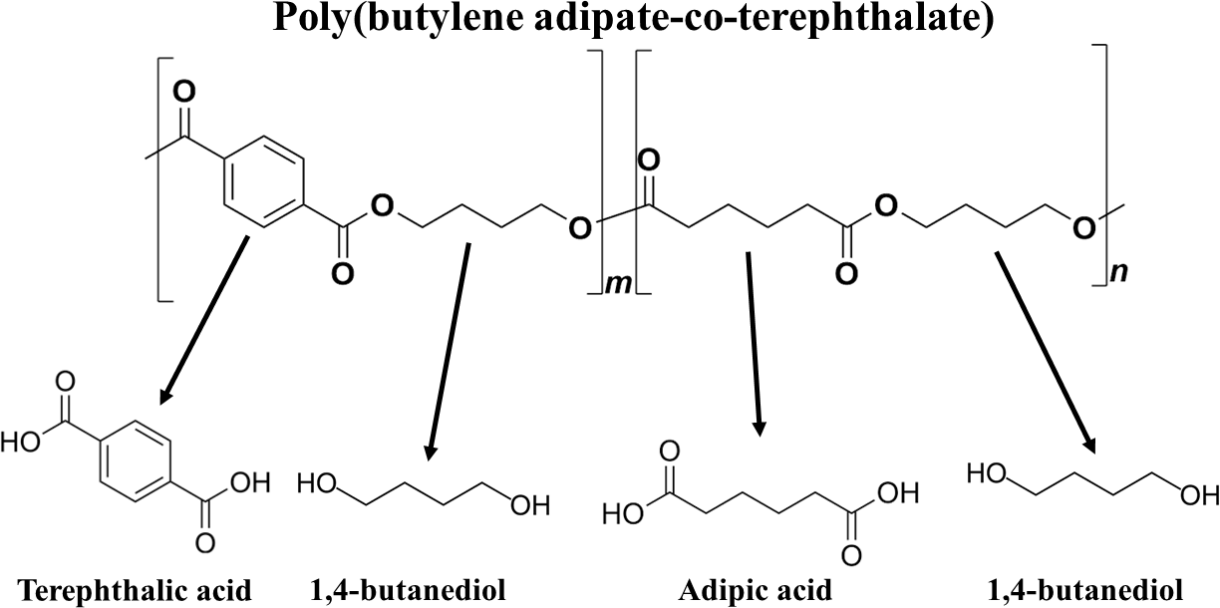
Molecular structure of PBAT and the corresponding by-products generated by FsCut through cleavage of the ester bonds linking its monomers: terephthalic acid, 1,4-butanediol, and adipic acid.

Poly(butylene adipate-co-terephthalate) (PBAT) is a long-chain aromatic–aliphatic copolyester composed of adipic acid, terephthalic acid (TPA), and 1,4-butanediol linked by ester bonds (Fig. 1). Its cost-effective, petroleum-based synthesis and favorable mechanical and thermal properties have made PBAT a widely used polymer. The copolymer combines the flexibility of aliphatic polyesters with the strength and thermal stability of aromatic components and can be processed by standard polymer manufacturing methods such as extrusion, injection molding, and film blowing. Consequently, PBAT is employed in packaging, agriculture, biomedical, and textile applications (1, 2).

In 2023, global PBAT production was approximately 279,000 tons, underscoring its low manufacturing cost and versatile physicochemical profile (3). However, its aromatic rings confer strong resistance to hydrolysis, making disposal and recycling challenging (4). Current PBAT recycling strategies include (i) mechanical recycling waste without altering its chemical structure, which can be repeated up to seven cycles with minimal thermal degradation (5); (ii) chemical recycling by depolymerizing PBAT into monomers or other valuable chemicals for repolymerization (6); and (iii) enzymatic degradation, biologically hydrolyzing the polymer into its monomeric constituents (7).

Among these methods, enzymatic degradation is advantageous because it takes place under mild conditions, reducing energy demand and avoiding the use of toxic reagents. Cutinases and related enzymes selectively cleave ester bonds in PBAT, releasing its monomers with no other undesirable by-products. In contrast, mechanical recycling gradually deteriorates polymer quality, and chemical recycling often generates unwanted side reactions (1, 8).

Although PBAT is classified as a biodegradable polymer, meaning it can be enzymatically degraded, PBAT exhibits an extremely slow degradation rate due to its aromatic ring, which hinders hydrolysis (Fig. 1). Under freshwater sediment conditions, PBAT exhibited minimal molecular-weight reduction and no complete degradation after 24 months (9). Isolated bacterial strains degraded only 12%–13% of PBAT mass after 8 weeks. PBAT displays the lowest biodegradation rate under aquatic conditions compared to other biodegradable polyesters, such as poly(3-hydroxybutyrate) (PHB), poly(3-hydroxybutyrate-co-3-hydroxyvalerate) (PHBV), poly(butylene succinate) (PBS), poly(ε-caprolactone) (PCL), and poly(lactic acid) (PLA) (10). Enhanced enzymatic PBAT hydrolysis occurs only under optimized conditions like engineered enzymes at 60°C and pH 9 buffers, which limit practical applicability due to energy and reagent costs (7).

Certain enzymes not specialized for PBAT depolymerization, including cutinases, lipases, and carboxylesterases, catalyze ester-bond (-COO-) hydrolysis due to their broad substrate specificity shared with natural esters present in biomolecules such as lipids (1, 11, 12). Among them, *Fusarium solani* cutinase (FsCut, E.C. 3.1.1.74) stands out for its broad specificity, accessible active site, catalytic efficiency, and stability across conditions. FsCut hydrolyzes both natural cutin and synthetic polyesters, including PBAT, and remains active across a broad range of pH (7–9) and temperature conditions (30°C–50°C) (13, 14). Its degradative activity was proven in previous studies, where *Escherichia coli* expressing FsCut released 0.518 mM TPA from ground PBAT after 7 days, significantly higher than AbEst and PsEst esterases (0.076 and 0.033 mM, respectively). Moreover, FsCut expression enabled *E. coli* growth on PBS as a sole carbon source, confirming its potential for bioremediation (13).

In this study, FsCut was heterologously expressed in *Paracoccus denitrificans* PD1222, a metabolically versatile, genetically tractable bacterium with a fully sequenced genome (15). *P. denitrificans* can utilize adipic acid and 1,4-butanediol (two PBAT monomers) as carbon sources. The species also synthesizes and stores PHB (16–18). PHB functions as a carbon and energy reservoir, being accumulated under carbon excess and mobilized during its limitation. Its thermoplasticity and renewable microbial synthesis make PHB promising for packaging, agricultural, and biomedical uses (19).

In the present work, *P. denitrificans* PD1222 was transformed with a plasmid (named pV1) specifically designed to express and secrete FsCut outside the cell in this species. This plasmid contains the strong constitutive promoter *P*tuf, the *fsCut* gene, and the PorG signal peptide. *P*tuf ensures robust transcription in *P. denitrificans* and other alphaproteobacteria (20). The *fsCut* sequence previously used in *E. coli* expression systems was employed for consistency (13). The PorG signal peptide mediates extracellular secretion of FsCut, facilitating contact with polymeric substrates (21, 22). Along with its construction and functionality, this synthetic expression vector was transformed directly into *P. denitrificans* using an improved protocol.

## MATERIALS AND METHODS

### Bacterial strains

Two bacterial strains were used in this study: *E. coli* DH5α and *P. denitrificans* PD1222. Chemically competent *E. coli* DH5α cells (23) were employed for plasmid propagation and transformation of the vectors of interest (pV1 and pV0). *P. denitrificans* PD1222 was kindly provided by Prof. Dr. Rob van Spanning (Vrije Universiteit Amsterdam) (16).

### Culture media and growth conditions

Luria–Bertani (LB) medium was used to culture both *E. coli* and *P. denitrificans* strains, as well as for experiments involving the latter. Liquid and solid LB media were prepared by dissolving 25 and 35 g/L of commercial LB powder, respectively, in distilled water. Media were sterilized by autoclaving at 121°C for 20 min. Cultures were incubated at 37°C, with liquid cultures shaken at 200 rpm.

A saline mineral medium (MSM) was also employed, following the formulation described by Coleman’s laboratory (24). The MSM contained 0.67 g (NH_4_)_2_SO_4_, 0.95 g KH_2_PO_4_, 2.27 g K_2_HPO_4_, and 1% (wt/vol) succinic acid dissolved in 900 mL of deionized water. The pH was adjusted to 7.0 with NaOH, and the volume was brought to 1 L. After autoclaving (121°C, 20 min) and cooling, the medium was supplemented with a 500× trace element solution. The composition of this 500× solution (per liter) was 6.37 g Na_2_EDTA·2H_2_O, 1 g ZnSO_4_·7H_2_O, 0.5 g CaCl_2_·2H_2_O, 2.5 g FeSO_4_·7H_2_O, 0.1 g NaMoO_4_·2H_2_O, 0.1 g CuSO_4_·5H_2_O, 0.2 g CoCl_2_·6H_2_O, 0.52 g MnSO_4_·H_2_O, and 60 g MgSO_4_·7H_2_O (24).

For PBAT degradation assays, 3 g of PBAT (M·VERA B5037; Bio-Fed, Köln, Germany) was suspended in 40 mL of MSM. PBAT was previously ground and sieved to obtain particles <100 µm in diameter and sterilized under ultraviolet light. *P. denitrificans* PD1222 carrying either pV1 or pV0 was cultured in this medium at 30°C and 200 rpm.

Selective antibiotics were added to prevent contamination and ensure growth of the target strains. Wild-type *P. denitrificans* PD1222 is intrinsically resistant to rifampicin, whereas plasmids pV1 and pV0 confer resistance to ampicillin and kanamycin. Accordingly, *E. coli* strains harboring either plasmid were cultured with ampicillin and kanamycin, while *P. denitrificans* PD1222 (pV1/pV0) was grown in the presence of all three antibiotics. Final concentrations were 100 µg/mL ampicillin, 50 µg/mL kanamycin, and 25 µg/mL rifampicin in both liquid and solid media.

All culture preparation and handling steps were performed under sterile conditions in a laminar flow cabinet.

### Restriction enzymes, DNA ligase, and Klenow polymerase

Restriction digestions with NsiI and XbaI (Thermo Fisher Scientific), ligations with T4 DNA ligase (Thermo Fisher Scientific), and end-filling reactions using the Klenow fragment (Thermo Fisher Scientific) were performed following the manufacturer’s protocols.

### Vectors

A synthetic expression vector (7,853 bp) was constructed for heterologous protein expression in *P. denitrificans* (Fig. S1). The plasmid comprises (i) the *repA* origin of replication from the pIND4 vector (1,020 bp), previously shown to support stable replication in a broad range of gram-negative bacteria, including *Paracoccus* species (25); (ii) a kanamycin resistance marker followed by the *rrnB* transcriptional terminator, which has been demonstrated to efficiently terminate transcription and reduce read-through effects in bacterial plasmids (26); and (iii) the *pUC57*-mini-BsaI-Free backbone, providing the *bla* gene and the *pUC* origin of replication, commonly used for high-copy number maintenance in *E. coli* prior to transfer to other hosts (GenBank accession no. PZ247360).

The expression cassette carries the *Fusarium solani* cutinase gene (Q99174), under the control of the constitutive *P*tuf promoter and fused to the *porG* signal peptide for efficient secretion, followed by transcriptional terminator sequences that have been validated to support proper transcript maturation and stability (21). The *P*tuf promoter has been previously confirmed as a strong constitutive element in *P. denitrificans*, ensuring high-level gene expression under standard growth conditions (20).

From plasmid pV1, a second vector was obtained through restriction digestion, called pV0 (Fig. S1), which lacked the cutinase gene and its promoter *P*tuf. This vector was used as a control during the experimentation. The pV0 plasmid (6,937 bp) was generated by enzymatic digestion of pV1 with NsiI and XbaI, followed by filling in the incompatible ends with the Klenow polymerase and subsequent religation with a T4 DNA ligase (GenBank accession no. PZ247361). The resulting plasmid contained all elements of pV1 explained above except for the cargo gene *fsCut* and the *P*tuf promoter.

### Transformation protocol in *E. coli* DH5α with pV1 and pV0

Chemically competent *E. coli* DH5α cells (23) were transformed separately with plasmids pV1 and pV0 by heat shock following the manufacturer’s protocol. Briefly, 50 µL of competent cells was mixed with 1 µL of plasmid DNA (40 ng/µL), heat-shocked, and incubated for 1 h before plating on LB agar containing ampicillin and kanamycin. Plates were incubated for 16 h, and resulting colonies were restreaked onto fresh antibiotic-containing plates.

### Transformation protocol in *P. denitrificans* PD1222 with pV1 and pV0

A simplified heat shock-based transformation protocol for *P. denitrificans* PD1222 was established based on a previously described method (27), with the aim of reducing procedural complexity. Chemically competent cells were generated using 0.1 M CaCl_2_ and 0.15 M MgCl_2_ solutions kept on ice, and cultures were grown under selective conditions to mid-exponential phase prior to competency induction. All subsequent handling steps were performed under cold conditions to preserve cell competency.

When the culture reached an OD_600_ of 0.8, cells were precooled to 4°C and centrifuged at 5,500 rpm for 5 min at 4°C. The supernatant was discarded, and the pellet was washed without resuspension using 10 mL of CaCl_2_. The pellet was then resuspended in 5 mL of CaCl_2_ and incubated on ice for 15 min. After centrifugation, the supernatant was removed and the pellet was resuspended in MgCl_2_. The cells were centrifuged again, the supernatant discarded, and the final pellet resuspended in 0.5 mL of CaCl_2_, obtaining a highly dense competent cell suspension (final OD_600_ ≈ 12). Aliquots of 50 µL were prepared and used immediately for transformation.

For plasmid uptake, aliquots were incubated with 10 µL of plasmid DNA (230.8 ng/µL for pV1 or 241.5 ng/µL for pV0) on ice for 10 min. This was followed by a heat-shock treatment adapted for this strain (39°C for 8 min), rapid chilling (30 s), and incubation in 250 µL of SOC medium at 37°C for 1 h. Cells were then allowed to recover prior to plating on selective agar containing rifampicin, kanamycin, and ampicillin. Transformants were obtained after 48 h of incubation.

### PCR for screening of transformant colonies

Colonies obtained after transformation of *E. coli* and *P. denitrificans* with pV1 and pV0 were screened by colony PCR using SapphireAmp Fast PCR Hot-Start Master Mix (Takara Bio). Reactions followed the manufacturer’s protocol (denaturation at 65°C, 15 s extension, 35 cycles).

Two primer pairs were used to verify plasmid presence: DMG01/DMG02 (5′-GGTGCGCCTGATCTTGGCGTCC-3′/5′-GCAAGCGGGGCGTCTTGACAACC-3′) amplified a 1,083 bp fragment of the *mobLS* region (Fig. S1), and Met-Dir-Check/Met-Rev-Check (5′-CCCATCAGCGTTGCTTAATTAATTGATGAC-3′/5′-CCCAGCACTAGTCCATGACGGCATGATTTC-3′) amplified either a 1,130 bp fragment in pV1 (Fig. S1) or a 214 bp fragment in pV0 (Fig. S1), confirming the presence or absence of the *fsCut* cargo gene.

For transformed *P. denitrificans* PD1222 colonies, an additional primer set (ntrB-dir/ntrB-rev) targeting the chromosomal *ntrB* gene (28) produced a 1,234 bp amplicon, confirming strain identity. The *ntrB* gene, which encodes a sensor kinase involved in nitrate assimilation under nitrogen-limiting conditions, was used as a gene target to prove that the host was *P. denitrificans* PD1222.

### RNA extraction and quantitative reverse transcription PCR analysis

*P. denitrificans* PD1222 strain harboring the pV1 plasmid was grown in 10 mL of LB or MSM supplemented with succinic acid at 37°C and 200 rpm. After 18 h, total RNA was extracted using the NZY Total RNA Isolation kit (NZYTech, cat. no. MB13402) according to the manufacturer’s instructions. To quantify the expression of the cutinase-encoding gene *fsCut* relative to the *rpoB* housekeeping gene, quantitative reverse transcription PCR (RT-qPCR) was performed.

The selection of *rpoB* as a reference gene for internal normalization in *P. denitrificans* PD1222 is based on previous studies that demonstrate its suitability for RT-qPCR normalization (29) and its presence as a single-copy gene in bacteria (30), which ensures accuracy in transcript quantification and normalization (31, 32).

Specific primers were designed for both the target and reference genes. The primer pair for *rpoB* (rpoBq-PD-Frw: 5′-TGCCGTATCATTCCCTATCGC-3′ and rpoBq-PD-Rev: 5′-GTTGTAGAAGGCATCCATGATGC-3′) yielded a 159 bp amplicon, while the *fsCut* primers (FsCutq-PD-Frw: 5′-CCGGTAATCTGGGTACGTTAGG-3′ and FsCutq-PD-Rev: 5′-AAGCATTTCACGGATCGCAG-3′) produced a 164 bp fragment. Both sets were designed with a melting temperature of 52°C.

The specificity of the primers was initially validated by conventional PCR using genomic DNA extracted with Genomic DNA Purification Kit (Thermo Fisher Scientific, cat. no. K0512) from the PD1222 pV1 strain as a template. The resulting products were analyzed via agarose gel electrophoresis, confirming the presence of a single, distinct band for each reaction and the absence of non-specific amplification.

Subsequent optimization was performed using cDNA synthesized from the total RNA of the PD1222 pV1 strain. Primer concentration assays were carried out to establish optimal performance, determining that a final concentration of 300 nM for both forward and reverse primers provided the highest amplification efficiency for both genes and both with a unique melt curve in each case (Fig. S2). This concentration for both primer pairs was used in the following assays. Furthermore, the amplification efficiency for each primer set was calculated using standard curves from serial dilutions of the template.

The RT-qPCR reactions were carried out using the TaqPath DuraPlex 1-Step RT-qPCR Master Mix (Applied Biosystems, cat. no. A58666) and supplemented with Green-Fluorescent DNA Stain (Jena Bioscience, cat. no. PCR-378) for fluorescence detection. All reactions followed the manufacturer’s recommendations. The hybridization time was 30 s at 52°C, while the amplification time was 30 s. Relative gene expression was assessed using the 2^−ΔCt^ method, and the fold change in expression was calculated according to the 2^−ΔΔCt^ method.

### PBAT assay in *P. denitrificans* PD1222 strains carrying pV1 or pV0

Twelve sterile 250 mL glass bottles were prepared, each containing 40 mL of culture medium: six with LB broth and six with MSM supplemented with 1% (wt/vol) succinic acid. After autoclaving, rifampicin, kanamycin, and ampicillin were added to all bottles at the appropriate concentrations, along with the trace element solution (only in bottles with MSM). In half of the bottles, 3 g of UV-sterilized PBAT granules (<100 µm) was included. *P. denitrificans* strains harboring pV1 or pV0 were precultured at 30°C with orbital shaking at 200 rpm in the corresponding media (LB or MSM) containing antibiotics until exponential phase (OD_600_ around 0.8), harvested by centrifugation (2,000 rpm, 5 min), and resuspended to an OD_600_ of 10.

Aliquots of 400 µL were then used to inoculate each bottle, resulting in an initial OD_600_ of 0.1. Cultures were incubated at 30°C and 200 rpm. All experimental conditions, including transformant strains and abiotic controls, were performed in triplicate as summarized in Table 1 and monitored over a 20-day period to quantify TPA release and assess growth dynamics.

**TABLE 1:** Different experimental conditions for testing *P. denitrificans* PD1222 strains harboring pV1 or pV0 with/without PBAT added^*a*^

Abiotic + PBAT	*P. denitrificans* PD1222 pV0 + PBAT	*P. denitrificans* PD1222 pV1 + PBAT	*P. denitrificans* PD1222 pV0	*P. denitrificans* PD1222 pV1
MSM + 1% succinic acid				
3 bottles	3 bottles	3 bottles	3 bottles	3 bottles
LB				
3 bottles	3 bottles	3 bottles	3 bottles	3 bottles

^*a*^ Triplicates were performed for each case.

Daily 1 mL samples were taken under sterile conditions for (i) OD_600_ measurements, (ii) TPA quantification from the supernatant after centrifugation, and (iii) assessment of polyhydroxyalkanoate (PHA) accumulation in the cell pellets.

### Extraction and isolation of PHA

Pellets obtained from the collected samples were pretreated to extract and isolate PHAs from the bacterial cells. Each pellet was resuspended in 1 mL of 1-propanol:HCl (80:20, vol/vol). After mixing, the suspension was transferred into Hach tubes. Subsequently, 2 mL of chloroform and 10 µL of benzoic acid (used as an internal standard with a stock solution of 40 g/L in 1-propanol) were added.

Standard solutions were prepared in parallel from a stock of PHB–PHV (88% PHB, 12% PHV) diluting the stock solution of 442 mg/L in chloroform. All Hach tubes were incubated in a digester at 100°C for 4 h. After incubation, the tubes were cooled to room temperature for 15 min, and 1 mL of molecular-grade water was added. After a 5-min resting period, two phases were formed: an aqueous phase and an organic phase. Since PHAs partitioned into the organic phase, this fraction was collected and filtered through 0.45 µm pore-size filters for subsequent analysis.

### Quantitative analysis of PHA

PHAs were analyzed by GC-MS using an Agilent 7820A GC coupled to an Agilent 5977E MSD. Separation was performed on a polar DB-WAX capillary column (30 m × 250 µm × 0.25 mm, polyethylene glycol coating) with helium (99.999%) as the carrier gas. Filtered organic extracts (1 µL) were injected at 1 mL/min and 7.1 psi. The oven was programmed from 40°C (5 min hold) to 200°C at 8°C/min (2 min hold), followed by a ramp to 240°C at 5°C/min, for a total runtime of 35 min. Retention times for PHB and PHV were 17.75 and 18.75 min, respectively (Fig. S3).

### Analytical method to determine TPA

As a result of FsCut-mediated PBAT hydrolysis in *E. coli* DH5α, monomers including TPA, adipic acid, and 1,4-butanediol were released (Fig. 1) (13). The supernatant from the PBAT degradation assays (see “RNA extraction and quantitative reverse transcription PCR analysis,” above) was used to quantify TPA, the only monomer not metabolized by *P. denitrificans* PD1222. Adipic acid and 1,4-butanediol can be metabolized and therefore excluded as degradation indicators (16, 33). The supernatant was adjusted to pH 3.5 with 2 M phosphoric acid, and the added acid was accounted for in the final calculations.

TPA was measured using an Alliance 2695 HPLC system with a refractive index detector equipped with a Phenomenex Synergi 4 µm Hydro-RP 80 Å column (150 × 4.6 mm). The flow rate was 0.6 mL/min under a gradient elution of molecular water, acetonitrile, and 10 mM H_2_SO_4_ at 25°C (Table 2).

**TABLE 2:** HPLC gradient to detect TPA molecules where it is mentioned in the flow and the respective buffers over time

Time (min)	Flow (mL/min)	Acetonitrile (%)	H_2_O (%)	10 mM H_2_SO_4_ (%)
0	0.6	18	62	20
20	0.6	18	62	20
21	0.6	90	10	0
26	0.6	90	10	0
27	0.6	18	62	20
30	0.6	18	62	20

The injection volume was 10 µL, with detection at 254 nm over 210–400 nm. Each run lasted 30 min, and TPA eluted at 9.703 min (Fig. S4).

### Plasmid stability assays

To evaluate the segregational stability of plasmids pV1 and pV0 in *P. denitrificans* PD1222, both engineered strains were separately cultured in LB or MSM supplemented with 1% succinic acid. Cultures were incubated at 37°C with orbital shaking at 200 rpm. Every 24 h, a subculture was performed by inoculating 200 µL of the previous culture into 10 mL of fresh medium, ensuring 20 consecutive days of growth in the absolute absence of antibiotic selection.

On day 20, plasmid maintenance was verified through two complementary methods. First, genomic DNA was extracted from the final cultures, and PCR was performed using the DMG01/DMG02 primer pair to target the 1,083 bp fragment of the plasmid backbone. Wild-type PD1222 was used as a negative control (Fig. S5). Second, a phenotypic assessment was conducted by spotting 4 µL of the day 20 cultures onto LB and MSM agar plates supplemented with either rifampicin alone or a combination of rifampicin, ampicillin, and kanamycin. The plates were incubated at 37°C for 24 h (Fig. S5).

## RESULTS

### Transformation efficiency of *P. denitrificans* PD1222

After incubation, transformants were obtained for both plasmids. For each plasmid, transformants were plated across three separate LB plates. In the case of pV1, a total of 104 colonies were recovered, distributed as 26, 35, and 43 colonies per plate. For pV0, a total of 137 colonies were obtained, distributed as 36, 38, and 53 colonies per plate. Colonies were subsequently confirmed by selective restreaking, excluding satellite colonies (34). Based on these values, transformation efficiencies were estimated at 45 CFU/µg DNA for pV1 (15 ± 3 CFU/µg DNA per plate) and 56 CFU/µg DNA for pV0 (17 ± 3 CFU/µg DNA per plate). Molecular screening by PCR of representative transformants (*n* = 10 per construct) revealed a positivity rate of 80% for both plasmids.

For comparison, transformations performed in chemically competent *E. coli* DH5α yielded 216 colonies for pV1 and 247 colonies for pV0, corresponding to transformation efficiencies of 5,400 and 6,175 CFU/µg DNA, respectively. PCR analysis confirmed plasmid presence in 100% of the analyzed *E. coli* colonies.

Transformation efficiency was calculated, taking into account the number of recovered colonies, DNA input, and plating and recovery volumes, with the formula


$$TE=\frac{N\;\times \;Vrec}{Vplate\;\times \;µg\;DNA}$$

To contextualize these results, a comparative analysis was conducted using data from previous conjugation assays performed in our laboratory with the same *P. denitrificans* PD1222 recipient strain and four vectors of comparable molecular weights to those used in this study. For this comparison, frequencies were defined as follows: transformation frequency (*f*_trans_) was calculated as the number of transformants per total competent cells used, while conjugation frequency (*f*_conj_) was determined as transconjugants per recipient cell (*P. denitrificans* PD1222 in all cases). Regarding pV0 (6,937 bp), the calculated *f*_trans_ (1.71·10^−6^) was compared to the mean *f*_conj_ of two similar vectors (both 7,150 bp), which was 1.54·10^−5^ ± 2.6·10^−6^. This indicates that the transformation of pV0-like plasmids achieves approximately 11.4% ± 2.64% of the conjugation frequency. Similarly, for pV1 (7,853 bp), the *f*_trans_ (1.3·10^−6^) was compared to the average *f*_conj_ of two plasmids of 8,384 bp, yielding 1.9·10^−5^ ± 7.6·10^−7^. In this case, the transformation frequency represented 6.9% ± 0.76% of the conjugation efficiency.

### Determination of TPA release by *P. denitrificans* PD1222 modified strains

**Fig 2:**
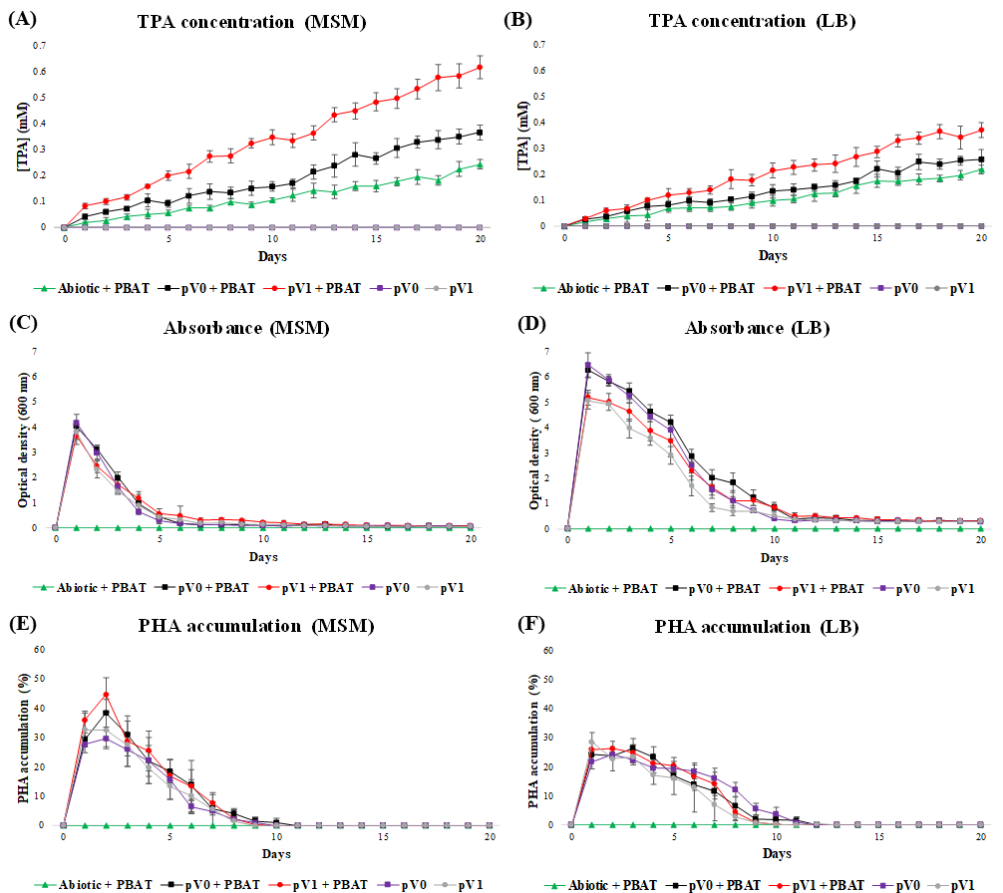
Terephthalic acid (TPA) concentration, cell growth, and PHA accumulation under different culture conditions. TPA concentration (mM) in MSM (**A**) and LB (**B**); cell growth measured as absorbance at 600 nm (OD_600_) in MSM (**C**) and LB (**D**); and percentage of PHA accumulation in MSM (**E**) and LB (**F**). Conditions include abiotic controls with PBAT (green triangles), *P. denitrificans* PD1222 pV0 with PBAT (black squares), PD1222 pV1 with PBAT (red circles), PD1222 pV0 without PBAT (purple squares), and PD1222 pV1 without PBAT (gray circles). Cultures were incubated at 30°C with orbital shaking at 200 rpm. All bottles were inoculated to an initial OD_600_ of 0.1, and 3 g of UV-sterilized PBAT granules (<100 µm) was added only to the indicated conditions.

As mentioned before, *P. denitrificans* PD1222 cannot metabolize TPA, making its accumulation in the medium a suitable marker for PBAT degradation (35, 36). TPA was detected only in cultures containing PBAT (Fig. 2A and B), while PD1222 strains without PBAT showed no TPA, as expected. Abiotic control with PBAT also exhibited TPA accumulation, likely due to environmental degradation by light and temperature (37–39). TPA was detected in the pV0 strain as well, possibly from hydrolytic activity of uncharacterized secreted enzymes.

TPA release was higher in MSM than in LB. On the final day, PD1222 carrying pV1 and pV0 released 0.618 ± 0.044 mM and 0.367 ± 0.028 mM TPA in MSM, and 0.37 ± 0.027 mM and 0.257 ± 0.037 mM in LB, respectively. As expected, the pV1 strain showed greater TPA accumulation than the one carrying the pV0 plasmid in both media.

Slopes of TPA release over time (Fig. 2A and B) were calculated for each strain and condition (Table 3). The contribution of FsCut was determined as the difference between the PD1222 pV1 and pV0 strains, while pV0 strain rates were corrected for abiotic degradation.

**TABLE 3:** Slopes of TPA and PBAT degradation curves in LB (green) and MSM (yellow) media under different culture conditions^*a*^

Slopes	mmol (TPA)/L/day	mol (PBAT)/s	mol (PBAT)/s/m^2^
LB medium			
Abiotic	0.98·10^−2^	1.81·10^−11^	1.23·10^−10^
pV0	1.24·10^−2^	2.29·10^−11^	1.55·10^−10^
pV1	1.83·10^−2^	3.38·10^−11^	2.29·10^−10^
pV1–pV0	0.59·10^−2^	1.09·10^−11^	7.41·10^−11^
pV0–abiotic	0.26·10^−2^	4.81·10^−12^	3.26·10^−11^
pV1–abiotic	0.85·10^−2^	1.57·10^−11^	1.17·10^−10^
MSM medium			
Abiotic	1.05·10^−2^	1.94·10^−11^	1.31·10^−10^
pV0	1.72·10^−2^	3.18·10^−11^	2.15·10^−10^
pV1	3·10^−2^	5.55·10^−11^	3.76·10^−10^
pV1–pV0	1.28·10^−2^	2.37·10^−11^	1.61·10^−10^
pV0–abiotic	0.67·10^−2^	1.24·10^−11^	8.41·10^−11^
pV1–abiotic	1.95·10^−2^	3.61·10^−11^	2.44·10^−10^

^*a*^ “Abiotic” denotes the slope of the TPA degradation line under abiotic conditions; pV0 denotes the slope obtained with PD1222 pV0; pV1 denotes the slope obtained with PD1222 pV1; pV1–pV0 denotes the difference between the pV1 and pV0 slopes; pV0–abiotic denotes the difference between the pV0 and abiotic slopes; and pV1–abiotic denotes the difference between the pV1 and abiotic slopes. The values in the mmol (TPA)/L/day column correspond to the slopes obtained from Fig. 2A and B. The values in the other columns are based on the PBAT release rate, considering that TPA is one of the four monomers constituting the polymer (Fig. 1). A culture volume of 40 mL was used in the experiments, and the calculated surface area of the added PBAT granules was 0.1475 m^2^, derived from the addition of 3 g of polymer with an average diameter of 100 μm.

The highest TPA release occurred in MSM, with PD1222 pV1 exhibiting a slope of 3·10^−2^ mmol/L/day, followed by PD1222 pV0 at 1.72·10^−2^ mmol/L/day. In MSM, the pV1 strain released 42.7% more TPA than the pV0, and 39% more than the same strain in LB (1.83·10^−2^ mmol/L/day). Differences between slopes yielded a maximum of 1.28·10^−2^ mmol/L/day for pV1 vs pV0 in MSM and 0.67·10^−2^ mmol/L/day for pV0 vs abiotic control.

The degradation rate of PBAT by pV1 relative to pV0, normalized to both time and polymer surface area, was determined to be 7.41·10^−11^ and 1.61·10^−10^ mol (PBAT)/s/m^2^ in LB and MSM media, respectively. In contrast, the difference between pV0 and abiotic conditions exhibited degradation rates of 3.26·10^−11^ and 8.41·10^−11^ mol (PBAT)/s/m^2^ under the same conditions. Accordingly, the degradation of PBAT mediated by the pV1 strain was 2.27-fold higher in LB and 1.9-fold higher in MSM compared to that observed for the pV0 strain.

### Growth and PHA accumulation in transformant *P. denitrificans* PD1222 strains

No growth was observed in abiotic controls (Fig. 2C and D). In biotic bottles, both pV1 and pV0 strains reached peak biomass at 24 h in LB and MSM, with the pV0 strain achieving slightly higher yields, likely due to the energy cost of FsCut production.

After 24 h, biomass declined in both media, more markedly in MSM. Similar growth patterns in MSM with or without PBAT indicate that once succinic acid is depleted, PBAT degradation is too slow to supply sufficient adipic acid or 1,4-butanediol for cellular maintenance. These results suggest that external carbon supplementation would be required at an industrial scale to sustain growth and enhance PBAT degradation.

After peak biomass, PHAs were progressively depleted, confirming that adipic acid and 1,4-butanediol from PBAT were insufficient to sustain growth. In MSM (Fig. 2E), PD1222 pV1 with PBAT reached a maximum PHA content of 44.63% dry weight at 48 h, compared with 38.38% for the pV0 strain, representing a 1.16-fold increase. In LB (Fig. 2F), PHA accumulation was lower, with the highest value (28.56% dry weight) observed in the pV1 strain without PBAT at 24 h. Across all conditions, PHA levels were fully consumed between days 9 and 11, and overall trends were similar among the four strains.

### Transcriptional analysis of *fsCut* expression in *P. denitrificans* PD1222 pV1

Quantitative RT-qPCR analysis confirmed the successful transcription of *fsCut* in *P. denitrificans* PD1222 pV1. Expression levels were normalized against the housekeeping gene *rpoB* using the 2^−ΔCt^ method, in which a lower (more negative) ΔCt value indicates a higher relative abundance of the target transcript. Based on this analysis, *fsCut* transcripts were 3.94 ± 0.03-fold and 2.58 ± 0.02-fold more abundant than the reference gene in MSM and LB media, respectively. The relative change in expression (fold change) between media was calculated using the 2^−ΔΔCt^ method, where a 1.36 ± 0.01-fold upregulation of *fsCut* expression was observed in MSM supplemented with succinic acid compared with LB medium.

### Features of the pV1 vector designed to enable the synthesis and export of FsCut for degrading PBAT in *P. denitrificans* PD1222

The expression cassette, containing the *Fusarium solani* cutinase gene (Q99174), was placed under the control of the constitutive *P*tuf promoter and fused to the porG signal peptide with appropriate transcriptional terminator sequences. Each component functioned as expected: the *P*tuf promoter drove strong and consistent expression in *P. denitrificans*, and the *porG* signal peptide enabled successful secretion of the cutinase (20, 21). Together, these results demonstrate that the vector design reliably supports replication stability, antibiotic selection, and heterologous protein expression and secretion in *P. denitrificans*.

Regarding maintenance of both vectors without selective pressure, segregational stability assays confirmed that both pV1 and pV0 were robustly maintained in *P. denitrificans* PD1222 for at least 20 days of continuous growth without antibiotic pressure. PCR analysis on day 20 successfully amplified the characteristic 1,083 bp backbone fragment in all transformants grown in both rich (LB) and minimal (MSM) media, whereas no amplification was detected in the wild-type strain (Fig. S5).

These results were further corroborated by phenotypic screening, where the transformant strains harvested after 20 days of non-selective growth retained full resistance to ampicillin and kanamycin. In contrast, the wild-type strain was unable to grow on these antibiotics (Fig. S5). These findings demonstrate that the pIND4-derived origin of replication ensures high plasmid stability in *P. denitrificans* regardless of the nutritional environment.

## DISCUSSION

Although the plasmids pV1 and pV0 encode conjugation genes, transformation was preferred due to its shorter duration and avoidance of intermediate *E. coli* strains, completing in a single day compared to ≥2 days for conjugation (27, 40). While transformation efficiency in *P. denitrificans* PD1222 remains lower than in chemo-competent *E. coli* (>10^6^ to 10^9^ CFU/µg DNA), a positivity rate of 80% was achieved, comparable to previous reports in *E. coli* (>90%) (41, 42) and higher than conjugation in *P. denitrificans* DYTN-1 (initially 33% and only 100% after optimization) (43).

Regarding PBAT depolymerization, diverse biological strategies have been explored to enhance its relatively low depolymerization rate. Engineered cutinases have demonstrated the ability to fully decompose PBAT films, yielding measurable amounts of TPA over 10 days (7). Similarly, *Stenotrophomonas* sp. YCJ1 achieved a lipase activity of 10.5 U/mL alongside a 10 wt% film loss (11), while *Peribacillus frigoritolerans* JZ1 exhibited 12.45% degradation under optimized conditions (9). At a microbiome level, Fernandes et al. (44) reported that 47% of PBAT was mineralized in soil after 180 days using PHB/PBAT bilayer films. Despite these advances, most studies omit plastic degradation rates as a function of the added surface area, making a direct comparison with the results presented in Table 3 difficult. Furthermore, the subsequent microbial valorization of these breakdown products remains a significant challenge. This difficulty is consistent with recent findings by Santos-Beneit et al. (45), who reported that PBAT hydrolysates supported markedly lower PHA accumulation (~6%) in *P. denitrificans* PD1222 than other polyesters such as PLA (~15%) or PHB (30%), likely due to lower carbon solubilization efficiencies and reduced bioavailability of its specific monomers.

Previous studies report PHA accumulation in *P. denitrificans* ranging from 16% to 50% of dry weight, depending on strain, medium, carbon source, and nitrogen limitation. For instance, PD1222 accumulated 36% PHB at 40 h using 1-butanol in nitrogen-limited mineral medium (45), while MSM with sodium succinate yielded 16.66% PHB (46). More recently, Santos-Beneit et al. (45) demonstrated that PD1222 can accumulate up to 30% of its cell dry weight as PHA when grown on hydrolysates of PHB and PHBV and 13.4% when using succinate as the sole carbon source (45).

In the present study, PD1222 pV1 with PBAT reached higher PHA levels during the first 48 h (Fig. 2E and F), likely due to increased carbon availability from PBAT degradation, and converged with pV0 strain levels at later times, while complete PHA consumption happened between days 8 and 10. Following PHA accumulation, it was higher in MSM than LB, consistent with phosphorus and nitrogen-limited conditions enhancing PHA synthesis (47, 48).

Several strategies could be pursued to improve PBAT degradation and to enable growth of engineered *P. denitrificans* on the released monomers, as discussed in recent literature. From a protein engineering perspective, the catalytic efficiency, substrate affinity, and stability of polyester-degrading enzymes such as cutinases can be enhanced through rational mutagenesis or directed evolution, approaches that have been shown to substantially improve polymer hydrolysis rates (49, 50). In parallel, strain engineering strategies could focus on introducing or optimizing metabolic pathways for the uptake and assimilation of PBAT-derived monomers, such as adipic acid and 1,4-butanediol, which currently do not support growth of the engineered strain. In contrast, recent findings indicate that the wild-type PD1222 strain is naturally capable of metabolizing both adipic acid and 1,4-butanediol as sole carbon sources, achieving PHA/biomass yields of 10.5% and 5.0%, respectively, although with low growth rates (below 0.05 h^−1^) (45). The importance of combining enzymatic depolymerization with metabolic integration of released monomers has been highlighted as a key requirement for effective plastic biodegradation and upcycling (1).

The transcriptional analysis revealed that *fsCut* expression in PD1222 pV1 is influenced by the nutritional environment, with a 1.36-fold increase in MSM supplemented with succinic acid compared to the complex LB medium. This increase may suggest that the availability of a defined carbon source allows for more coordinated metabolic activity, potentially reducing the metabolic load often associated with heterologous protein production in complex media such as LB (16). Furthermore, cutinases like FsCut are enzymes whose heterologous expression often requires consistent carbon sources like succinate in the case of *P. denitrificans* PD1222, with the aim of supplying energy to support efficient transcription and translation (13).

Thus, MSM-succinate medium enhanced the transcriptional output of the *fsCut* gene compared to standard complex media, although the PBAT depolymerization rates were relatively similar between the two media, being 2.27-fold higher in LB and 1.9-fold higher in MSM compared to the pV0 strain. The slightly higher depolymerization rate observed in LB, notwithstanding its lower *fsCut* transcript levels, may be attributed to the metabolic background of *P. denitrificans* PD1222 in complex media. LB is a protein-rich environment that likely induces the synthesis and secretion of endogenous proteases.

### Conclusions

The transformation of *P. denitrificans* PD1222 with the pV1 plasmid was demonstrated to be a direct and rapid method. Although this transformation approach is approximately one order of magnitude less efficient than conjugation, the protocol yields a sufficient number of clones for routine genetic engineering while significantly reducing procedural time. This advantage makes it an advantageous alternative to traditional conjugation methods. Once established, the pV1 plasmid was stably maintained, enabling the successful expression and secretion of the target protein, FsCut. Transcriptional analysis confirmed this success, showing higher specific expression of fsCut in MSM compared to LB. Additionally, long-term stability assays revealed that the pIND4-derived replication origin ensures consistent segregational persistence of the vectors for up to 20 days without antibiotic selection, regardless of the nutritional complexity of the growth medium.

The functional efficiency of the system was evidenced by the PBAT depolymerization rates. Both the pV0 control strain and the FsCut-expressing strain exhibited depolymerizing activity. However, the pV1 strain achieved degradation rates 2.27-fold higher in LB and 1.9-fold higher in MSM relative to the pV0 strain.

Furthermore, the pV1 strain demonstrated a 1.16-fold increase in PHA accumulation at 24 h, suggesting that the products of PBAT depolymerization are being channeled into the strain’s carbon storage metabolism. Nevertheless, the current level of monomer release was insufficient to fully sustain bacterial growth in the absence of additional carbon sources. This highlights a common limitation in PBAT biodegradation and points toward the need for further optimization, such as additional genetic modifications or protein engineering of the FsCut enzyme, to enhance substrate uptake and metabolic integration.

## Data Availability

The plasmid sequences for pV0 and pV1 described in this study have been deposited in the GenBank database under accession numbers PZ247361 and PZ247360, respectively.

## SUPPLEMENTAL MATERIAL

Supplemental material aem.00358-26-s0001.docx Figures S1 to S5.

ASM does not own the copyrights to Supplemental Material that may be linked to, or accessed through, an article. The authors have granted ASM a non-exclusive, world-wide license to publish the Supplemental Material files. Please contact the corresponding author directly for reuse.

## References

[1] Martín-González D, de la Fuente Tagarro C, De Lucas A, Bordel S, Santos-Beneit F. 2024. Genetic modifications in bacteria for the degradation of synthetic polymers: a review. Int J Mol Sci 25:1–22. doi:10.3390/ijms25105536 PMC11121894 38791573

[2] Olonisakin K, Mohanty AK, Thimmanagari M, Misra M. 2025. Recent advances in biodegradable polymer blends and their biocomposites: a comprehensive review. Green Chem 27:11656–11704. doi:10.1039/D5GC01294E

[3] Ghasemlou M, Barrow CJ, Adhikari B. 2024. The future of bioplastics in food packaging: an industrial perspective. Food Packaging and Shelf Life 43:101279. doi:10.1016/j.fpsl.2024.101279

[4] Bher A, Mayekar PC, Auras RA, Schvezov CE. 2022. Biodegradation of biodegradable polymers in mesophilic aerobic environments. Int J Mol Sci 23:12165–12271. doi:10.3390/ijms232012165 36293023 PMC9603655

[5] Nomadolo N, Mtibe A, Ofosu O, Mekoa C, Letwaba J, Muniyasamy S. 2024. The effect of mechanical recycling on the thermal, mechanical, and chemical properties of poly (Butylene Adipate-Co-Terephthalate) (PBAT), Poly (Butylene Succinate) (PBS), Poly (Lactic Acid) (PLA), PBAT-PBS Blend and PBAT-TPS Biocomposite. J Polym Environ 32:2644–2659. doi:10.1007/s10924-023-03151-y

[6] Zheng WZ, Li X, Xie J, Zhang ZY, Wang PL, Huang D, Ren ZL, Ji JH, Wang GX. 2024. Closed-loop recycling of biodegradable poly(butylene adipate-co-terephthalate) based on hydrolysis and repolymerization strategy. J Environ Chem Eng 12:114354. doi:10.1016/j.jece.2024.114354

[7] Yang Y, Min J, Xue T, Jiang P, Liu X, Peng R, Huang JW, Qu Y, Li X, Ma N, Tsai FC, Dai L, Zhang Q, Liu Y, Chen CC, Guo RT. 2023. Complete bio-degradation of poly(butylene adipate-co-terephthalate) via engineered cutinases. Nat Commun 14:1645–1653. doi:10.1038/s41467-023-37374-3 36964144 PMC10039075

[8] Parodi A, Arpaia V, Samorì C, Mazzocchetti L, Galletti P. 2023. Novel strategies for recycling poly(butylene adipate- co -terephthalate)-starch-based plastics: selective solubilization and depolymerization–repolymerization processes. ACS Sustainable Chem Eng 11:14518–14527. doi:10.1021/acssuschemeng.3c03588

[9] Wufuer R, Li W, Wang S, Duo J. 2022. Isolation and degradation characteristics of PBAT film degrading bacteria. IJERPH 19:17087. doi:10.3390/ijerph192417087 36554967 PMC9779299

[10] García-Depraect O, Lebrero R, Rodriguez-Vega S, Bordel S, Santos-Beneit F, Martínez-Mendoza LJ, Aragão Börner R, Börner T, Muñoz R. 2022. Biodegradation of bioplastics under aerobic and anaerobic aqueous conditions: Kinetics, carbon fate and particle size effect. Bioresour Technol 344:126265. doi:10.1016/j.biortech.2021.126265 34737051

[11] Jia H, Zhang M, Weng Y, Zhao Y, Li C, Kanwal A. 2021. Degradation of poly(butylene adipate-co-terephthalate) by Stenotrophomonas sp. YCJ1 isolated from farmland soil. J Environ Sci (China) 103:50–58. doi:10.1016/j.jes.2020.10.001 33743918

[12] Wu P, Li Z, Gao J, Zhao Y, Wang H, Qin H, Gu Q, Wei R, Liu W, Han X. 2023. Characterization of a PBAT degradation carboxylesterase from Thermobacillus composti KWC4. Catalysts 13:340. doi:10.3390/catal13020340

[13] Santos-Beneit F, Chen LM, Bordel S, Frutos de la Flor R, García-Depraect O, Lebrero R, Rodriguez-Vega S, Muñoz R, Börner RA, Börner T. 2023. Screening enzymes that can depolymerize commercial biodegradable polymers: heterologous expression of Fusarium solani cutinase in Escherichia coli. Microorganisms 11:1–18. doi:10.3390/microorganisms11020328 PMC9963400 36838293

[14] Martinez C, De Geus P, Lauwereys M, Matthyssens G, Cambillau C. 1992. Fusarium solani cutinase is a lipolytic enzyme with a catalytic serine accessible to solvent. Nature 356:615–618. doi:10.1038/356615a0 1560844

[15] KEGG. 2025. Paracoccus denitrificans PD1222 – Complete genome (Entry T00440). KEGG. Available from: https://www. https://www.kegg.jp/entry/gn%3AT00440. Retrieved 22 Oct 2025.

[16] Bordel S, Martín-González D, Börner T, Muñoz R, Santos-Beneit F. 2024. Genome-scale metabolic model of the versatile bacterium Paracoccus denitrificans Pd1222. mSystems 9:e0107723. doi:10.1128/msystems.01077-23 38180324 PMC10878069

[17] Mothes G, Ackermann J ‐U, Babel W. 2004. Mole fraction control of Poly([R]‐3‐hydroxybutyrate‐co‐3‐hydroxyvalerate) (PHB/HV) Synthesized by Paracoccus denitrificans. Eng Life Sci 4:247–251. doi:10.1002/elsc.200320029

[18] Bordel Sergio, van Spanning RJM, Santos-Beneit F. 2021. Imaging and modelling of poly(3-hydroxybutyrate) synthesis in Paracoccus denitrificans. AMB Express 11:113. doi:10.1186/s13568-021-01273-x 34370106 PMC8353029

[19] Adnan M, Siddiqui AJ, Ashraf SA, Snoussi M, Badraoui R, Alreshidi M, Elasbali AM, Al-Soud WA, Alharethi SH, Sachidanandan M, Patel M. 2022. Polyhydroxybutyrate (PHB)-based biodegradable polymer from agromyces indicus: enhanced production, characterization, and optimization. Polymers (Basel) 14:3982. doi:10.3390/polym14193982 36235929 PMC9571180

[20] Schada von Borzyskowski L, Remus-Emsermann M, Weishaupt R, Vorholt JA, Erb TJ. 2015. A set of versatile brick vectors and promoters for the assembly, expression, and integration of synthetic operons in Methylobacterium extorquens AM1 and other alphaproteobacteria. ACS Synth Biol 4:430–443. doi:10.1021/sb500221v 25105793

[21] Saxena K, Richter OM, Ludwig B, Benz R. 1997. Molecular cloning and functional characterization of the Paracoccus denitrificans porin. Eur J Biochem 245:300–306. doi:10.1111/j.1432-1033.1997.00300.x 9151957

[22] Baker SC, Ferguson SJ, Ludwig B, Page MD, Richter O-MH, van Spanning RJM. 1998. Molecular genetics of the genus Paracoccus: metabolically versatile bacteria with bioenergetic flexibility. Microbiol Mol Biol Rev 62:1046–1078. doi:10.1128/MMBR.62.4.1046-1078.1998 9841665 PMC98939

[23] Thermo Fisher Scientific. 2025. DH5α competent cells (Cat. No. EC0112). Thermo Fisher Scientific. Available from: https://www.thermofisher.com/order/catalog/product/EC0112. Retrieved 22 Oct 2025.

[24] Coleman Lab. 2019. MSM medium (updated 2019). Coleman Laboratory, Michigan State University. Available from: https://coleman-lab.org/wp-content/uploads/2019/09/MSM-medium-updated-2019.pdf. Retrieved 22 Oct 2025.

[25] Ind AC, Porter SL, Brown MT, Byles ED, de Beyer JA, Godfrey SA, Armitage JP. 2009. Inducible-expression plasmid for Rhodobacter sphaeroides and Paracoccus denitrificans. Appl Environ Microbiol 75:6613–6615. doi:10.1128/AEM.01587-09 19684165 PMC2765152

[26] Simons RW, Houman F, Kleckner N. 1987. Improved single and multicopy lac-based cloning vectors for protein and operon fusions. Gene 53:85–96. doi:10.1016/0378-1119(87)90095-3 3596251

[27] Spence DW, Barr GC. 1981. A method for transformation of Paracoccus denitrificans. FEMS Microbiol Lett 12:159–161. doi:10.1111/j.1574-6968.1981.tb07632.x

[28] Olaya-Abril A, Hidalgo-Carrillo J, Luque-Almagro VM, Fuentes-Almagro C, Urbano FJ, Moreno-Vivián C, Richardson DJ, Roldán MD. 2018. Exploring the denitrification proteome of Paracoccus denitrificans PD1222. Front Microbiol 9:1137. doi:10.3389/fmicb.2018.01137 29896187 PMC5987163

[29] de Oliveira PAA, Baboghlian J, Ramos COA, Mançano ASF, Porcari A de M, Girardello R, Ferraz LFC. 2024. Selection and validation of reference genes suitable for gene expression analysis by reverse transcription quantitative real-time PCR in Acinetobacter baumannii. Sci Rep 14:3830. doi:10.1038/s41598-024-51499-5 38360762 PMC10869792

[30] Bivand JM, Dyrhovden R, Sivertsen A, Tellevik MG, Patel R, Kommedal Ø. 2024. Broad-range amplification and sequencing of the rpoB gene: a novel assay for bacterial identification in clinical microbiology. J Clin Microbiol 62:e0026624. doi:10.1128/jcm.00266-24 38884485 PMC11324016

[31] Stoddard SF, Smith BJ, Hein R, Roller BRK, Schmidt TM. 2015. rrnDB: improved tools for interpreting rRNA gene abundance in bacteria and archaea and a new foundation for future development. Nucleic Acids Res 43:D593–8. doi:10.1093/nar/gku1201 25414355 PMC4383981

[32] Větrovský T, Baldrian P. 2013. The variability of the 16S rRNA gene in bacterial genomes and its consequences for bacterial community analyses. PLoS One 8:e57923. doi:10.1371/journal.pone.0057923 23460914 PMC3583900

[33] Ren M, Li D, Addison H, Noteborn WEM, Andeweg EH, Glatter T, de Winde JH, Rebelein JG, Lamers MH, Schada von Borzyskowski L. 2025. NAD-dependent dehydrogenases enable efficient growth of Paracoccus denitrificans on the PET monomer ethylene glycol. Nat Commun 16:1–15. doi:10.1038/s41467-025-61056-x 40592898 PMC12214560

[34] Yurtsev EA, Chao HX, Datta MS, Artemova T, Gore J. 2013. Bacterial cheating drives the population dynamics of cooperative antibiotic resistance plasmids. Mol Syst Biol 9:1–8. doi:10.1038/msb.2013.39 PMC3779801 23917989

[35] Sasoh M, Masai E, Ishibashi S, Hara H, Kamimura N, Miyauchi K, Fukuda M. 2006. Characterization of the terephthalate degradation genes of Comamonas sp. strain E6. Appl Environ Microbiol 72:1825–1832. doi:10.1128/AEM.72.3.1825-1832.2006 16517628 PMC1393238

[36] Gautom T, Dheeman D, Levy C, Butterfield T, Alvarez Gonzalez G, Le Roy P, Caiger L, Fisher K, Johannissen L, Dixon N. 2021. Structural basis of terephthalate recognition by solute binding protein TphC. Nat Commun 12:6244. doi:10.1038/s41467-021-26508-0 34716322 PMC8556258

[37] Gutiérrez-Silva K, Capezza AJ, Gil-Castell O, Badia-Valiente JD. 2025. UV-C and UV-C/H₂O-induced abiotic degradation of films of commercial PBAT/TPS blends. Polymers (Basel) 17:1–29. doi:10.3390/polym17091173 PMC12073353 40362957

[38] Kanwal A, Zhang M, Sharaf F, Li C. 2022. UV-C and UV-C/H₂O-induced abiotic degradation of films of commercial PBAT/TPS blends. Polymers (Basel) 17:1–29. doi:10.3390/polym17091173 PMC12073353 40362957

[39] Ye H, Li Q, Li J, Li D, Ao Z. 2025. Review on the abiotic degradation of biodegradable plastic poly(butylene adipate-terephthalate): mechanisms and main factors of the degradation. Chinese Chemical Letters 36:109861. doi:10.1016/j.cclet.2024.109861

[40] Simon R, Priefer U, Pühler A. 1983. A broad host range mobilization system for in vivo genetic engineering: transposon mutagenesis in gram negative bacteria. Nat Biotechnol 1:784–791. doi:10.1038/nbt1183-784

[41] Yang Y, Yu Q, Wang M, Zhao R, Liu H, Xun L, Xia Y. 2022. Escherichia coli BW25113 competent cells prepared using a simple chemical method have unmatched transformation and cloning efficiencies. Front Microbiol 13:1–12. doi:10.3389/fmicb.2022.838698 PMC8989280 35401484

[42] Jacobus AP, Gross J. 2015. Optimal cloning of PCR fragments by homologous recombination in Escherichia coli*.* PLoS One 10:e0119221. doi:10.1371/journal.pone.0119221 25774528 PMC4361335

[43] Shi Y, Cao W, Zheng Z, Xu S, Chai L, Zhou S, Deng Y. 2024. Identification and characterization of an R-M system in Paracoccus denitrifican DYTN-1 to improve the plasmid conjugation transfer efficiency. J Microbiol Biotechnol 34:1826–1835. doi:10.4014/jmb.2402.02041 39155392 PMC11473606

[44] Fernandes M, Salvador AF, Vicente AA. 2024. Biodegradation of PHB/PBAT films and isolation of novel PBAT biodegraders from soil microbiomes. Chemosphere 362:142696. doi:10.1016/j.chemosphere.2024.142696 38925517

[45] Santos-Beneit F, Bordel S, Martín-González D, de la Fuente C, García-Depraect O, Börner T, Börner RA, Muñoz R. 2026. Valorization of polyester wastes into polyhydroxyalkanoates via a one-step microbial fermentation process. Process Biochem 161:283–289. doi:10.1016/j.procbio.2025.12.019

[46] Kojima T, Nishiyama T, Maehara A, Ueda S, Nakano H, Yamane T. 2004. Expression profiles of polyhydroxyalkanoate synthesis-related genes in Paracoccus denitrificans. J Biosci Bioeng 97:45–53. doi:10.1016/S1389-1723(04)70164-4 16233588

[47] Silva F, Campanari S, Matteo S, Valentino F, Majone M, Villano M. 2017. Impact of nitrogen feeding regulation on polyhydroxyalkanoates production by mixed microbial cultures. N Biotechnol 37:90–98. doi:10.1016/j.nbt.2016.07.013 27457131

[48] Guerra-Blanco P, Cortes O, Poznyak T, Chairez I, García-Peña EI. 2018. Polyhydroxyalkanoates (PHA) production by photoheterotrophic microbial consortia: effect of culture conditions over microbial population and biopolymer yield and composition. Eur Polym J 98:94–104. doi:10.1016/j.eurpolymj.2017.11.007

[49] Tournier V, Duquesne S, Guillamot F, Cramail H, Taton D, Marty A, André I. 2023. Enzymes’ power for plastics degradation. Chem Rev 123:5612–5701. doi:10.1021/acs.chemrev.2c00644 36916764

[50] Sui B, Wang T, Fang J, Hou Z, Shu T, Lu Z, Liu F, Zhu Y. 2023. Recent advances in the biodegradation of polyethylene terephthalate with cutinase-like enzymes. Front Microbiol 14:1265139. doi:10.3389/fmicb.2023.1265139 37849919 PMC10577388

