# Supplementary material for: Engineering Paracoccus denitrificans PD1222 for Fusarium solani cutinase-mediated biodegradation of poly(butylene adipate-co-terephthalate)
Source: Appl Environ Microbiol. 2026 Jun 18;92(7):e00358-26. doi: 10.1128/aem.00358-26 (PMC13390341; doi:10.1128/aem.00358-26)
Supplement: Supplemental material — Figures S1 to S5. [file aem.00358-26-s0001.docx]

**SUPPLEMENTAL FIGURES**

**FIG S1** Schematic representation of plasmids pV0 and pV1. **(A)** Map of pV0 (6,937 bp), generated by enzymatic digestion of pV1 with NsiI and XbaI, followed by filling in of the incompatible ends with Klenow polymerase and religation using T4 DNA ligase. The construct retains all pV1 elements except for the cargo gene *fsCut* and its promoter *P*tuf. **(B)** Map of the synthetic expression vector pV1 (7,853 bp), harboring the cargo gene fsCut under the control of the *P*tuf promoter and fused to the porG signal peptide sequence. In both maps, oligonucleotide pairs DMG01/DMG02 and Met-Dir-Check/Met-Rev-Check are indicated, along with the restriction sites for NsiI and XbaI used in the cloning strategy**.**


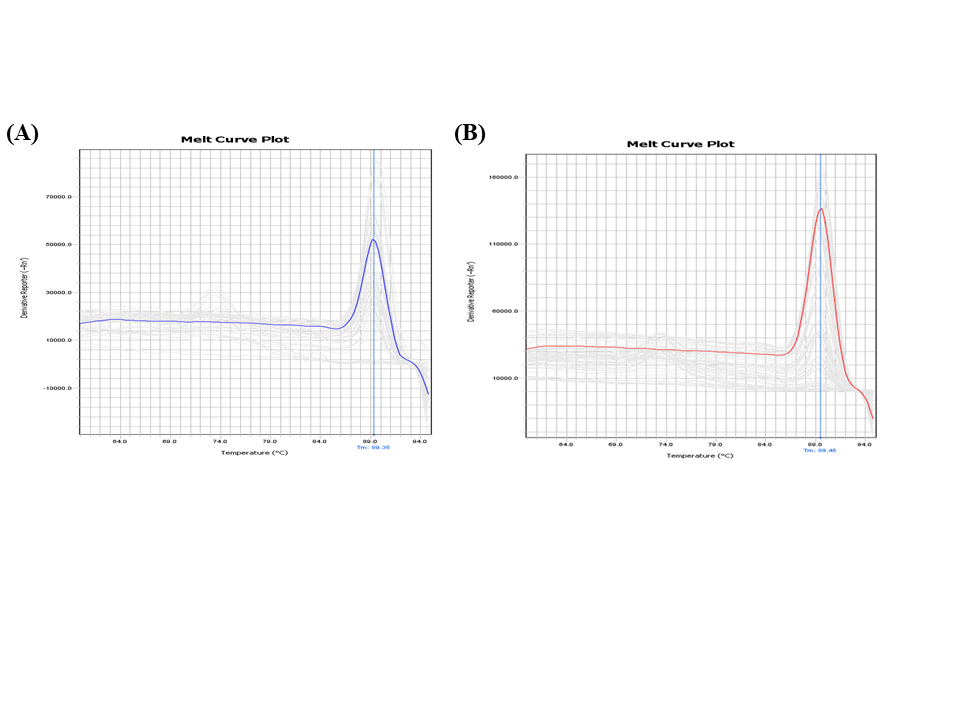


**FIG S2** Melting curves generated by RT-qPCR corresponding to the *rpoB* (**A**, blue) and *fsCut* (**B**, red) genes, showing the specificity of each primer pair (rpoBq-PD-Frw and rpoBq-PD-Rev for the *rpoB* gene, and FsCutq-PD-Frw and FsCutq-PD-Rev for the *fsCut* gene). The images correspond to melting curve analyses in which the final concentration of both primers in the reaction mix was 300 nM.


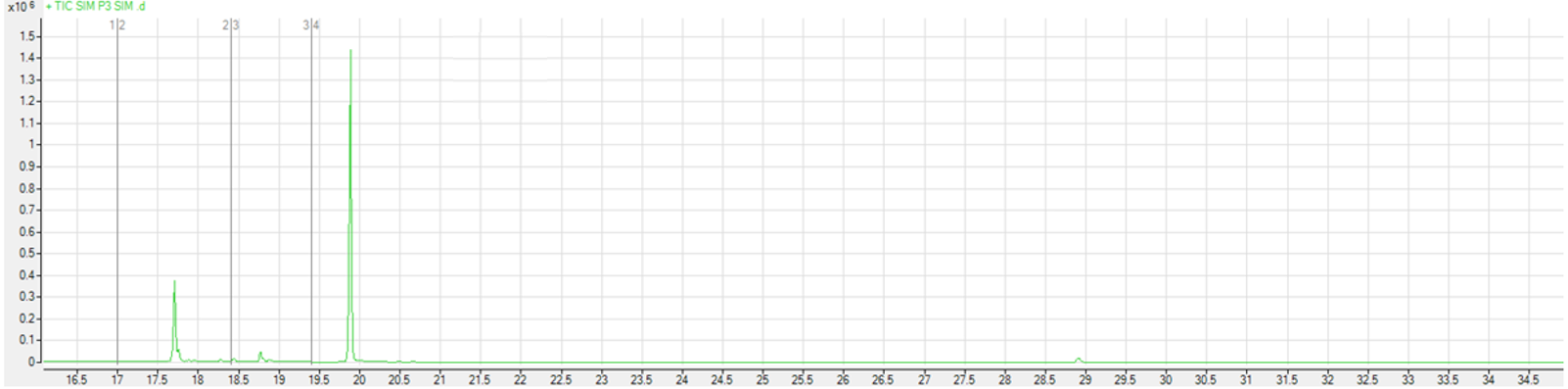


**FIG S3** PHA standard peaks and its retention times from the stock solution (442 mg/L; 88% PHB, 12% PHV), where the peak on the left refers to PHBs (17.75 min), the middle one to PHVs (18.75 min) and the one on the right to benzoic acid (19.88 min). GC-MS was used to measure. The analysis was performed by GC-MS, with an Agilent 7820A GC coupled to an Agilent 5977E MSD. Separation was carried out using a polar DB-WAX capillary column (30 m × 250 µm × 0.25 mm, polyethylene glycol coating) with pure helium as the carrier gas. Filtered standards and organic extracts (1 µL) were injected at 1 mL/min and 7.1 psi. The oven was programmed from 40 °C (5 min hold) to 200 °C at 8 °C/min (2 min hold), followed by a ramp to 240 °C at 5 °C/min, for a total runtime of 35 min.


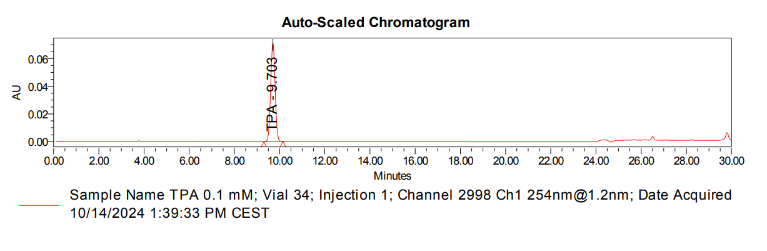


**FIG S4** Retention time of a TPA standard (9.703 min) using an Alliance 2695 HPLC system with a refractive index detector, equipped with a Phenomenex Synergi 4 µm Hydro-RP 80 Å column (150 × 4.6 mm). The flow rate was 0.6 mL/min under a gradient elution of molecular water, acetonitrile, and 10 mM H_2_SO_4_ at 25 °C, referred in Table 2 in text. The injection volume was 10 µL, with detection at 254 nm over 210–400 nm.

**FIG S5** Segregational stability and phenotypic characterization of plasmids pV0 and pV1 in *P. denitrificans* PD1222. **(A)** PCR-based verification of plasmid maintenance after 20 days of continuous growth without antibiotic pressure. DNA was extracted from cultures subcultured daily in LB and MSM media supplemented with 1% of succinic acid. Wells A–C (LB) and D–F (MSM) correspond to *P. denitrificans* harboring pV0, pV1, and the wild-type strain, respectively. The presence of the characteristic 1,083 bp backbone fragment in all transformant samples, and its absence in the WT negative control, confirms the persistence of the vectors. **(B)** Phenotypic screening for antibiotic resistance markers. Cultures from day 20 were spotted onto LB and MSM agar plates supplemented with rifampicin (Rf) as a baseline, or a cocktail of rifampicin, ampicillin, and kanamycin (Rf Amp Km). For each plate, the spots represent PD1222 pV0 (top left), PD1222 pV1 (top right), and WT (bottom).
